# Pulmonary Hypertension in Patients with Chronic Fibrosing Idiopathic Interstitial Pneumonias

**DOI:** 10.1371/journal.pone.0141911

**Published:** 2015-12-02

**Authors:** Marius M. Hoeper, Juergen Behr, Matthias Held, Ekkehard Grunig, C. Dario Vizza, Anton Vonk-Noordegraaf, Tobias J. Lange, Martin Claussen, Christian Grohé, Hans Klose, Karen M. Olsson, Thomas Zelniker, Claus Neurohr, Oliver Distler, Hubert Wirtz, Christian Opitz, Doerte Huscher, David Pittrow, J. Simon R. Gibbs

**Affiliations:** 1 Department of Respiratory Medicine and German Center of Lung Research (DZL), Hannover Medical School, Hannover, Germany; 2 Department of Internal Medicine V, University of Munich, Munich, Germany; 3 Department of Internal Medicine, Respiratory Medicine and Cardiology, Mission Medical Hospital, Würzburg, Germany; 4 University Hospital Heidelberg, Heidelberg, Germany; 5 Department of Cardiovascular and Respiratory Diseases, Sapienza, University of Rome, Rome, Italy; 6 Department of Pulmonary Diseases, VU University Medical Center, Amsterdam, The Netherlands; 7 Department of Internal Medicine II, Division of Pneumology, University Medical Center Regensburg, Regensburg, Germany; 8 LungenClinic, Grosshansdorf, Germany; 9 Department of Respiratory Medicine, ELK Thorax Centre, Berlin, Germany; 10 University Medical Center Hamburg-Eppendorf, Center of Oncology, Department of Respiratory Medicine, Hamburg, Germany; 11 Department of Cardiology, Angiology and Pneumology, University of Heidelberg, Heidelberg, Germany; 12 Division of Rheumatology, University Hospital Zurich, Zurich, Switzerland; 13 Department of Respiratory Medicine, University of Leipzig, Leipzig, Germany; 14 Department of Cardiology, DRK Kliniken Berlin Köpenick, Berlin, Germany; 15 Department of Rheumatology and Clinical Immunology, Charité University Hospital, and Epidemiology unit, German Rheumatism Research Centre, Berlin, Germany; 16 Institute for Clinical Pharmacology, Medical Faculty, Technical University, Dresden, Germany; 17 Department of Cardiology, National Heart & Lung Institute; Imperial College London, London, United Kingdom; Nippon Medical School Graduate School of Medicine, JAPAN

## Abstract

**Background:**

Pulmonary hypertension (PH) is a common finding in patients with chronic fibrosing idiopathic interstitial pneumonias (IIP). Little is known about the response to pulmonary vasodilator therapy in this patient population. COMPERA is an international registry that prospectively captures data from patients with various forms of PH receiving pulmonary vasodilator therapies.

**Methods:**

We retrieved data from COMPERA to compare patient characteristics, treatment patterns, response to therapy and survival in newly diagnosed patients with idiopathic pulmonary arterial hypertension (IPAH) and PH associated with IIP (PH-IIP).

**Results:**

Compared to patients with IPAH (n = 798), patients with PH-IIP (n = 151) were older and predominantly males. Patients with PH-IIP were treated predominantly with phosphodiesterase-5 inhibitors (88% at entry, 87% after 1 year). From baseline to the first follow-up visit, the median improvement in 6MWD was 30 m in patients with IPAH and 24.5 m in patients with PH-IIP (p = 0.457 for the difference between both groups). Improvements in NYHA functional class were observed in 22.4% and 29.5% of these patients, respectively (p = 0.179 for the difference between both groups). Survival rates were significantly worse in PH-IIP than in IPAH (3-year survival 34.0 versus 68.6%; p<0.001). Total lung capacity, NYHA class IV, and mixed-venous oxygen saturation were independent predictors of survival in patients with PH-IIP.

**Conclusions:**

Patients with PH-IIP have a dismal prognosis. Our results suggest that pulmonary vasodilator therapy may be associated with short-term functional improvement in some of these patients but it is unclear whether this treatment affects survival.

**Trial Registration:**

clinicaltrials.gov NCT01347216

## Introduction

The term idiopathic interstitial pneumonia (IIP) describes a large and heterogeneous group of inflammatory and fibrotic lung diseases [1]. According to the current classification, the major IIPs are grouped into chronic fibrosing IIPs, which include idiopathic pulmonary fibrosis (IPF) and idiopathic nonspecific interstitial pneumonia (NSIP), smoking-related IIPs, and acute/subacute IIPs [1]. Chronic fibrosing IIPs are by far the most common entities in this group of disease. Despite well-established diagnostic criteria, a clear distinction between IPF and NSIP is not always possible [1].

Pulmonary hypertension (PH), defined by a mean pulmonary arterial pressure (PAPm) ≥ 25 mmHg at rest, is a common complication of chronic fibrotic IIPs [2–4]. In patients with IPF, the prevalence of PH ranges from 8% up to 85% depending on the stage and severity of the underlying disease [5–7]. The prevalence of PH in patients with NSIP is less well studied. The development of PH in patients with IIP is associated with deterioration in exercise capacity and is an important predictor of mortality [8–13]. A number of drugs from various classes (endothelin receptor antagonists [ERA], phosphodiesterase-5 inhibitors [PDE5i], prostacyclin analogues, and soluble guanylate cyclase stimulators) have been approved for the treatment of pulmonary arterial hypertension (PAH) [14]. It is unknown, however, whether treatment with these pulmonary vasodilators affect symptoms and outcomes in patients with IIP. This question has not been addressed by large-scale randomized controlled trials, and preliminary studies have yielded conflicting results [15, 16]. Nevertheless, patients suffering from PH associated with IIP (PH-IIP) are occasionally treated with pulmonary vasodilators, especially when they present with severe haemodynamic impairment. There is yet no approved therapy for patients with PH-IIP, while consensus statements recommend that patients with severe PH should be referred to expert centres, noting that treatment with pulmonary vasodilators may be justified in selected cases [3, 17].

Given the lack of robust evidence from randomised clinical trials, important information may be derived from registries that prospectively enrol and systematically follow such patients. COMPERA (*Comparative*, *Prospective Registry of Newly Initiated Therapies for Pulmonary Hypertension*) is a large-scale international registry that collects data from patients with various forms of PH receiving targeted medical therapies. In the present study, we utilized the COMPERA database to analyse patient characteristics, demographics, treatment patterns, response to therapy and survival in patients with PH-IIP. Patients with idiopathic PAH (IPAH) were chosen as controls since the short-term and long-term responses to PAH targeted therapies are well known in this patient population.

## Methods

### Setting and participants

COMPERA was launched in July 2007 and continues to enrol patients (study identifier: clinicaltrials.gov NCT01347216). The registry was initially designed to capture data from patients with pulmonary arterial hypertension treated with endothelin receptor antagonists. After Amendment 2, which became active on June 1^st^, 2009, COMPERA enrolled patients with all forms of PH on any pulmonary vasodilator therapy. As of March 2015, 62 PH centres from 9 countries (Germany, Belgium, Netherlands, Italy, Austria, Switzerland, United Kingdom, Australia, Japan) participated, with 83% of the patients coming from Germany. Documentation includes demographics (age, gender), type of PH according to the Dana Point classification, date of the initial cardiac catheterization, New York Heart Association functional class, 6 min walk distance (6MWD), selected laboratory variables including arterial blood gas analyses, haemodynamics, pulmonary function test (PFT) data, and detailed information about medications used to treat PH.

The participating centres enter eligible patients on a consecutive basis. Data are collected at the time of diagnosis (baseline), and at least in 6-monthly intervals or whenever the patient has a predefined clinical event (death, transplantation, PH-related hospitalization, deterioration in functional class, any unscheduled change in PAH therapy, or other serious adverse events). Out-of-range data or missing values are automatically queried during data entry and source data verification is ensured by independent onsite monitoring.

Inclusion criteria for the present analysis were a diagnosis of IPAH or PH-IIP, age ≥ 18 years, and availability of data from right heart catheterization at diagnosis showing a mean pulmonary artery pressure (PAPm) ≥25 mmHg and a mean pulmonary arterial wedge pressure (PAWP) ≤ 15 mmHg. The diagnoses of IPAH and PH-IIP were made by the participating investigators based on current guidelines [1, 18]. The classification of the underlying lung disease was based on multidisciplinary assessments by pneumologists, pathologists and radiologists. Surgical lung biopsies were available from 25% of the patients with PH-IIP. We included patients diagnosed with IPAH or PH-IIP between January 1^st^, 2009, and June 1^st^, 2014. The cut-off date for follow-up assessments was March 11^th^, 2015. Death was ascertained according to country specific methods using databases in the healthcare system.

In order to ensure a strictly incident patient population, only patients entered into the database within 6 months after PH diagnosis were eligible for the present analysis.

COMPERA has been approved by the institutional review boards of Hannover Medical School, University of Munich, Mission Medical Hospital Würzburg, University of Heidelberg, University of Rome, University of Amsterdam, University of Regensburg, LungenClinic Großhansdorf, ELK Thorax Center and DRK Klinikum Berlin, University of Leipzig, University of Zurich and Technical University of Dresden (leading institutional review board). Written informed consent was obtained from all participating patients.

### Statistical analysis

Categorical data were displayed as number of patients (n) and respective relative frequency. Frequency distributions between the groups were compared with the χ^2^-test or Fisher test, respectively. For continuous data, normally distributed data were displayed as mean and standard deviation, otherwise median and Q1 to Q3 were shown with Q1 being the 25^th^ percentile and Q3 being the 75^th^ percentile. Group differences for normally distributed data were tested with a 2-sided t-test; otherwise, 2-sided Mann-Whitney test was used. The clinical response to therapy was assessed by changes in 6 min walking distance (6MWD) and functional class from baseline to the first follow-up assessment. Various combinations of changes in functional class and 6MWD changes were explored to identify short-term treatment responses that may be relevant in terms of predicting long-term outcome. Subgroup analyses were performed for PH-IIP patients who presented at baseline with a PAPm ≥35 mmHg or a PAPm ≥25 mmHg with a cardiac index (CI) below 2.0 L/min/m^2^, as these variables had been proposed to define severe PH in patients with lung disease during the last World Symposium on Pulmonary Hypertension [3]. Survival was compared using Kaplan-Meier estimates and Log-Rank-testing. All survival analyses were done from the time of PH diagnosis, i.e. the time of the first right heart catheterization. Patients lost to follow-up were censored at the time of the last visit. For analysis of factors associated with survival in PH-IIP, single predictor Cox regression analyses based on all variables listed in Table 1 were followed by multivariable Cox regression analysis including all the variables, which were statistically significant at the univariate level of p<0.1 except for PaO_2_ at room air and serum levels of N-terminal fragment of pro-brain natriuretic peptide (NT-proBNP), which were omitted due to more than 25% missing values. P-values <0.05 were considered significant; no adjustment was done for multiple testing.

**Table 1:** Patient demographics and baseline characteristics.

	IPAH (n = 798)	PH-IIP (n = 151)	p value
Female (n, %)	478 (59.9%)	56 (37.1%)	<0.001
Age, years (mean, SD)	64.5±15.8	71.1±10.7	<0.001
BMI, kg/m^2^ (mean/SD)	28.0±6.7	26.9±5.0	0.033
6MWD, m (mean, SD)	303±129	251±116	<0.001
WHO Class I (n, %)	3 (0.4%)	0	*
WHO Class II (n, %)	98 (12.5%)	4 (2.7%)	*
WHO Class III (n, %)	568 (72.4%)	106 (71.6%)	*
WHO Class IV (n, %)	115 (14.7%)	38 (25.7%)	*
TLC (% pred)	95.3±17.2	68.8±17.0	<0.001
FVC (% pred)	82.1±21.6	62.9±20.0	<0.001
FEV_1_ (% pred)	76.7±21.4	67.7±20.2	0.001
DLCO (% pred)	50.1±20.5	28.5±15.8	<0.001
paO_2_ (mmHg)^a^	62.4±13.4	56.3±10.4	<0.001
paCO_2_ (mmHg)^a^	34.5±7.1	37.4±5.5	0.001
RAP, mmHg	8.6±5.0	5.9±4.8	<0.001
mPAP, mmHg	45±13	37±9	<0.001
PAWP, mmHg	9.6±3.4	8.0±3.8	<0.001
PVR, dyn.s.cm^-5^	793±433	649±268	0.001
CI, L/min/m^2^	2.2±0.7	2.1±0.6	0.438
SvO_2_, %	63±9	64±8	0.063
Bilirubin (mg/dl)	0.7 (0.5–1.0)	0.6 (0.5–0.9)	0.011
Creatinine (mg/dl)	1.0 (0.8–1.3)	1.0 (0.8–1.3)	0.755
Uric acid (mg/dl)	7.2 (5.7–9.0)	7.1 (5.7–8.3)	0.224
NT-proBNP (ng/L)	1,627 (577–3,487)	1,029 (373–2901)	0.065
BNP (ng/L)	233 (93–469)	114 (59–256)	0.009

All baseline variables were more than 90% complete except for PaO_2_ at room air and serum levels of BNP/NT-proBNP.

Abbreviations: IPAH, idiopathic pulmonary arterial hypertension; PH-IIP, pulmonary hypertension associated with idiopathic interstitial pneumonia; 6MWD, 6 min walking distance; BMI, body mass index; WHO, world health organization; TLC, total lung capacity; FVC, forced vital capacity; FEV_1_, forced expiratory capacity in one second; DLCO, diffusion capacity of the lung for carbon monoxide; RAP, right atrial pressure; PAPm, mean pulmonary arterial pressure; PAWP, pulmonary arterial wedge pressure; CI, cardiac index, SvO_2_, mixed venous oxygen saturation; PVR, pulmonary vascular resistance; mixed-venous oxygen saturation; BNP, brain natriuretic peptide; NT-proBNP, N-terminal fragment of pro-brain natriuretic peptide; Q1, 25^th^ percentile; Q3, 75^th^ percentile; pred, predicted normal value;

^a^room air measurements only

*p<0.001 for all WHO functional classes combined

## Results

This study enrolled 798 patients with IPAH and 151 patients with PH-IIP (IPF, n = 113; NSIP, n = 38). The median follow-up time was 24.1 months in the IPAH population and 13.2 months in the PH-IIP population. Table 1 summarizes the clinical and hemodynamic characteristics of the two populations at the time of PH diagnosis. Compared with IPAH patients, PH-IIP patients were older and more often male. The majority of patients in both groups presented in an advanced WHO functional class, and patients with PH-IIP were more likely to present in functional class IV. Pulmonary function was well preserved in patients with IPAH whereas patients with PH-IIP showed the expected restrictive ventilation pattern; 35.3% of these patients had a forced vital capacity (FVC) ≥70% of the predicted value. The diffusion capacity for carbon monoxide and the arterial pO_2_ were significantly lower in patients with PH-IIP than in patients with IPAH. The average haemodynamic profile of patients with PH-IIP was that of severe pulmonary hypertension with impaired right ventricular function, indicated by a mean pulmonary artery pressure (PAPm) of 37±9 mmHg, a pulmonary vascular resistance (PVR) of 649±268 dyn·s·cm^-5^, and a cardiac index of 2.1±0.6 L/min/m^2^. The current criteria for severe PH (PAPm ≥35 mmHg or PAPm ≥25 mmHg with CI <2.5 l/min/m^2^) were met in 79% of these patients.

### Use of pulmonary vasodilators and early response to therapy

According to the inclusion criteria, all patients received pulmonary vasodilator therapies at inclusion, but the selected drugs differed substantially between the two patient populations (Table 2). At entry, 79% of the patients with IPAH received monotherapy with either an ERA or a PDE5i, while 15% received initial combination therapy. In contrast, 95% of the patients with PH-IIP received monotherapy, in most (88%) of the cases, a PDE5i. After 1 year, 57% of the patients with IPAH were receiving monotherapy with either an ERA or a PDE5i, while 39% were receiving combination therapies. In contrast, 87% of the patients with PH-IIP received monotherapy with a PDE5i and only 2% received combination therapy.

**Table 2:** PH targeted therapy at inclusion and 1 year after diagnosis.

	IPAH (n = 798)	PH-IIP (n = 151)	p value
**Baseline**			
ERA monotherapy	172 (21.6%)	11 (7.3%)	<0.001
PDE-5 inhibitor monotherapy	461 (57.8%)	133 (88.1%)	<0.001
PCA monotherapy	13 (1.6%)	0	t.n.a.
Other monotherapy	29 (3.6%)	0	t.n.a.
ERA+PDE-5 inhibitor	85 (10.7%)	4 (2.6%)	0.001
Other double combination therapies	29 (3.6%)	3 (2.0%)	0.459
**1 year**			
ERA monotherapy	72 (13.6%)	4 (4.7%)	0.020
PDE-5 inhibitor monotherapy	228 (43.1%)	75 (87.2%)	<0.001
PCA monotherapy	0	1 (1.2%)	t.n.a.
Other monotherapy	8 (1.5%)	0	t.n.a.
ERA+PDE-5 inhibitor	129 (24.4%)	2 (2.3%)	<0.001
Other double combination therapies	42 (7.9%)	0	0.002
Triple combination therapy	33 (6.2%)	0	t.n.a.
No therapy	17 (3.2%)	4 (4.7%)	t.n.a.

Abbreviations: IPAH, idiopathic pulmonary arterial hypertension; PH-IIP, pulmonary hypertension associated with idiopathic interstitial pneumonia; ERA, endothelin receptor antagonist; PDE5, phosphodiesterase-5; PCA, prostacyclin analogue; t.n.a, test not applicable

The cumulative drug discontinuation rates were similar in both groups. ERA were discontinued in 21% of the IPAH patients and in 18% of the PH-IIP patients; PDE5i were discontinued in 11% of the IPAH patients and in 12% of the PH-IIP patients. For both classes of drugs and in both patient populations, lack of clinical improvement and lack of tolerability were the predominant reasons for drug discontinuations (data not shown).

The median interval from baseline to the first follow-up visit was 13 weeks in both groups. At first follow-up in the IPAH group, the median improvement in 6MWD was 30 m in patients with IPAH and 24.5 m in patients with PH-IIP (p = 0.457). Overall, the changes in 6MWD from baseline to the first follow-up visits were comparable in both groups (Table 3).

**Table 3:** Changes in 6 min walking distance (6MWD) from baseline to the first follow-up visit in patients with idiopathic pulmonary arterial hypertension (IPAH) and patients with pulmonary hypertension associated with idiopathic interstitial pneumonia (PH-IIP).

	Total	IPAH	PH-IIP	p
6MWD improvement ≥20 m	58.8%	59.5%	54.2%	0.530
6MWD improvement ≥30 m	52.7%	53.8%	45.8%	0.353
6MWD improvement ≥40 m	44.5%	45.9%	35.4%	0.213
6MWD improvement ≥50 m	35.7%	36.4%	31.3%	0.522
Worsening in 6MWD	26.6%	26.3%	29.2%	0.726
No change in 6MWD (change 0–19 m)	14.6%	14.2%	16.6%	0.661

Functional class improved in 29.5% of the patients with IPAH; 65.5% remained unchanged and 5.0% worsened. In patients with PH-IIP, functional class improved in 22.4%, remained unchanged in 71.4% and worsened in 6.1% (p = 0.356 compared to IPAH).

We compared the response to treatment in patients with PH-IIP who did and did not fulfil the criteria of severe PH. There was no significant difference in the proportion of patients who improved their 6MWD or their functional class, respectively, between these two groups (changes in 6MWD grouped in 10 m-steps from 20 m to 50 m; all p>0.6; data not shown).

### Survival and predictors of outcome

**Fig 1:**
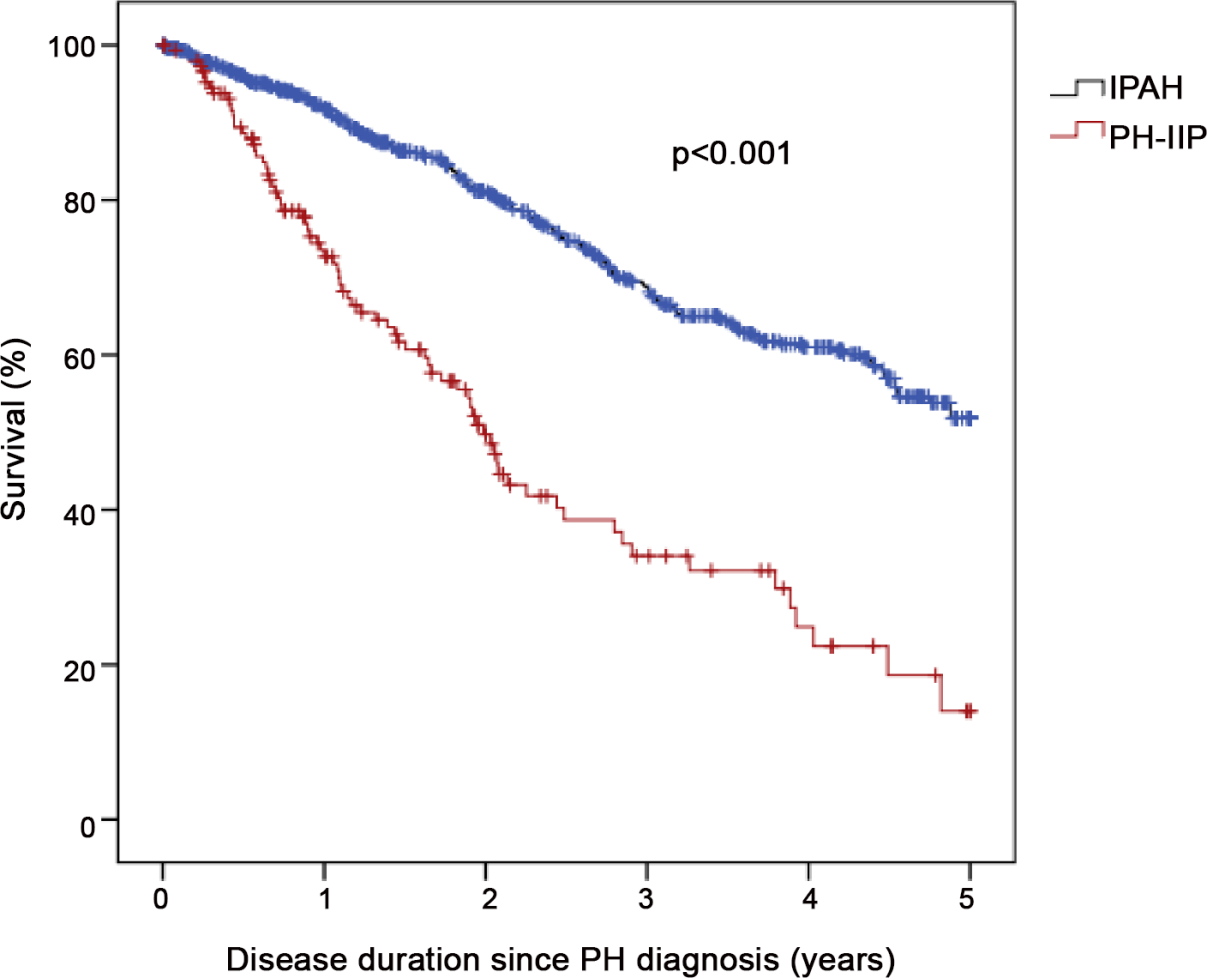
Kaplan-Meier survival estimates in patients with pulmonary hypertension associated with chronic fibrosing idiopathic interstitial pneumonias (PH-IIP) and patients with idiopathic pulmonary arterial hypertension (IPAH). Numbers at risk at baseline and after 1 year, 2 years, 3 years, 4 years and 5 years in the IPAH cohort were 786, 558, 382, 253, 154 and 43, respectively, and in the PH-IIP cohort 150, 84, 40, 21, 10 and 2, respectively.

Thirteen patients (1.4%; 12 IPAH, 1 PH-IIP) were not eligible for survival analysis since only baseline documentation was available. The survival status could be almost completely ascertained except for 21 (2.2%) patients who were lost to follow-up; 17 (2.2%) in the IPAH group and 4 (2.7%) in the PH-IIP group. These patients were censored at their last follow-up visit. During the 5-year follow-up, there were 284 deaths; 207 deaths occurred in patients with IPAH (crude mortality rate, 26.3%) and 77 deaths occurred in patients with PH-IIP (crude mortality rate, 51.3%). The estimated survival probabilities at 1, 3 and 5 years in the IPAH group were 91.8%, 68.6% and 51.8%, and 72.7%, 34.0% and 14.0%, respectively, in the PH-IIP group (p<0.0001 by log-rank analysis; Fig 1). The leading cause of death in the IPAH group was right heart failure (58.9%). In the PH-IIP group, right heart failure accounted for 28.6 of all fatalities, while the leading cause of death was respiratory failure (49.1%). There was no difference in survival between patients with PH due to IPF and patients with PH due to NSIP (p = 0.115; data not shown). Survival was, however, worse in patients with more severely impaired lung function. When we divided the PH-IIP patient population according to the median FVC of 62% predicted, patients with a lower FVC had a higher mortality risk (p = 0.047; data not shown).

In the univariate Cox regression analysis of baseline variables potentially predicting outcome among PH-IIP patients, factors associated with a poor survival were NYHA class IV, age, 6MWD, mixed venous oxygen saturation (SvO_2_), total lung capacity (TLC), PaO_2_, and NT-proBNP (p<0.1). None of the hemodynamic variables and no lung function variables other than TLC were predictors of outcome. In multivariable analysis NYHA class IV, SvO_2_, and TLC remained significantly associated with survival (Table 4).

**Table 4:** Univariate and multivariate Cox proportional hazard analysis of risk factors for death in patients with PH-IIP.

	Univariate model HR (95% CI)	P value	Multivariate model HR (95% CI)	P value
NYHA IV	2.122 (1.288–3.496)	0.003	1.626 (1.211–2.183)	0.001
Age* (years)	1.118 (0.989–1.264)	0.075		
6-MWD^#^ (m)	0.958 (0.934–0.983)	0.001		
SvO_2_ ^$^ (%)	0.774 (0.658–0.910)	0.002	0.712 (0.577–0.880)	0.002
TLC^$^ (% pred.)	0.934 (0.868–1.005)	0.067	0.888 (0.819–0.963)	0.004
PaO_2_ (mmHg)^§^	0.873 (0.755–1.008)	0.065		
NT pro-BNP (pg/ml)^&^	1.01 (1.002–1.019)	0.016		

Estimated hazard ratios (HR), 95% confidence intervals (CI) and p-values were calculated by Cox regression analyses. SvO_2_ denotes mixed venous oxygen saturation; 6MWD, 6 min walking distance; NYHA, New York Heart Association.

* per 5-years increase

# per 10m increase

$ per 5% increase

§ per 5 mmHg increase

& per 100 pg/ml increase

**Fig 2:**
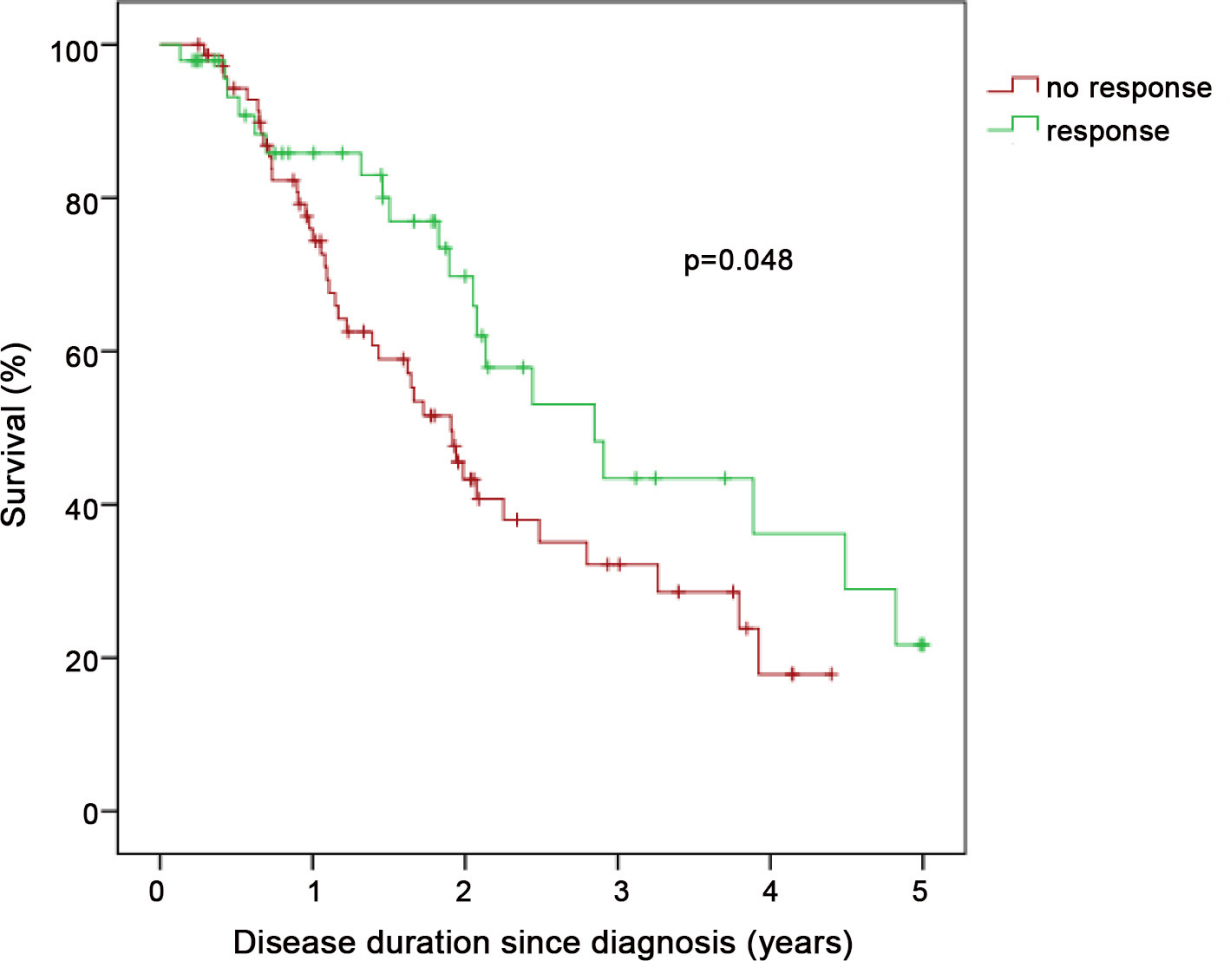
Kaplan-Meier survival estimates in patients with pulmonary hypertension associated with chronic fibrosing idiopathic interstitial pneumonias (PH-IIP) stratified by clinical response at first follow-up defined as either improvement in 6 min walking distance by at least 20 m or improvement in NYHA functional class. Numbers at risk at baseline and after 1 year, 2 years, 3 years, 4 years and 5 years in the “no response” cohort were 73, 50, 22, 11, 3 and 0, respectively, and in the “response” cohort were 48, 28, 15, 8, 5 and 2, respectively.

In patients with PH-IIP, an improvement in 6MWD by at least 20 m or in functional class at the first follow-up visit was associated with a better survival compared to patients who did not met these criteria, despite almost identical haemodynamics at baseline. In the “responders”, the survival rates at 1, 2, and 3 years were 84.4%, 66.2% and 41.7%, respectively. The respective survival rates in the “non-responders” were 75.8%, 46.8%, and 33.9%, respectively (p = 0.048 by log-rank analysis; Fig 2). Similar trends were seen with different cut-offs levels for 6MWD improvements of ≥30 m, ≥40 m and ≥50 m, but these were not statistically significant (data not shown). There was no difference in survival between PH-IIP patients whose 6MWD improved less or more than 20% from baseline (p = 0.879).

## Discussion

The present paper describes a cohort of patients with chronic fibrotic IIPs and associated PH. These patients were treated with pulmonary vasodilators, mostly PDE5i, and the proportion of patients with a short-term response to therapy as indicated by improvements in 6 min walking distance or functional class was comparable to what was found in patients with IPAH. However, the outcome of patients with PH-IIP was dismal with survival rates of 72.7%, 34.0% and 14.0%, after 1, 3 and 5 years, respectively. NYHA class IV, SvO_2_, and TLC were independently associated with a high mortality risk.

It is unclear whether PH-targeted therapies affect the outcomes of patients with PH-IIP as suggested by preliminary data from Japan [19]. Due to the lack of a control group, our data do not allow us to draw such conclusions. We found that PH-IIP who responded to treatment with an improvement in 6MWD by at least 20 m or an improvement in functional class tended to have a better survival. A similar observation has been made by Hurdman and co-workers in patients with chronic obstructive pulmonary disease and PH [20]. Still, restraint is required when interpreting these data, which should be considered not more than hypothesis generating.

The observed outcomes in our series are in line with previous publications and underscore the catastrophic prognosis of patients with PH-IIP. Lettieri et al. described similar survival rates in 79 patients with IPF and PH, none of who received PH-targeted therapies [10]. In an analysis from the ASPIRE (Assessing the Spectrum of Pulmonary Hypertension Identified at a Referral Centre) registry, the 3-year survival of 32 patients with IIP-PH was 16% [21]; which was even worse than in our series. These patients had similar baseline characteristics as the patients in our cohort, but it is unclear how many of them received treatment with pulmonary vasodilators. In a recent study, Brevis et al. compared responses to pulmonary vasodilator therapy and survival in patients with PH due to various lung diseases and patients with IPAH [22]. This study contained 22 patients with PH due to interstitial lung disease (PH-ILD) with baseline characteristics comparable to our series. The response to pulmonary vasodilator therapy in these patients was less pronounced than in patients with IPAH; NT-proBNP improved significantly, 6MWD improved by 20 m (not statistically significant) and functional class was unchanged. The 3-year survival rate of the PH-ILD patients was approximately 20%, which was, again, worse than in our series.

Very few randomized controlled trials have assessed the effects of pulmonary vasodilator therapies in patients with PH-IIP. There have been several studies addressing the hypothesis that ERA might be effective in treating pulmonary fibrosis, three with bosentan and one each with ambrisentan and macitentan, but none of them found positive effects [23–27]. The ARTEMIS-IPF (placebo-controlled study to evaluate safety and effectiveness of ambrisentan in idiopathic pulmonary fibrosis) trial was even terminated early because of an increased rate of disease progression and respiratory hospitalizations in patients receiving ambrisentan [26]. Of note, all of these studies targeted pulmonary fibrosis, not pulmonary hypertension in patients with pulmonary fibrosis. The only randomized controlled trial to date specifically addressing the use of ERA in patients with PH-IIP was recently published by Corte and co-workers [15]. In this study, patients with PH-IIP were 2:1 randomized to bosentan or placebo. The primary endpoint was the change in the pulmonary vascular resistance index after 16 weeks. The study enrolled 60 patients, but only 39 were available for a second right heart catheterization. There were no significant effects of bosentan on haemodynamics, symptoms, functional class, or deaths.

The overall negative studies on ERA in patients with ILD with or without PH may explain why ERA were rarely used to treat PH-IIP in our series, while almost 90% of these patients received PDE5i. However, there is also little evidence that PDE5i are safe and effective in patients with PH-IIP. The STEP-IPF (Sildenafil Trial on Exercise Performance in Idiopathic Pulmonary Fibrosis) study addressed the use of sildenafil in patients with IPF [16]. The patients enrolled in this study suffered from more advanced pulmonary fibrosis than the patients in our series. STEP-IPF included only patients with a low diffusion capacity for carbon monoxide (<35% of the predicted value) in order to select patients with a high likelihood of having concomitant PH. Data from right heart catheterization were not available. The study failed to meet its primary endpoint, the proportion of patients with ≥20% improvement in 6MWD after 12 weeks of therapy. This endpoint was met in 10% of the patients in the sildenafil group and in 7% of the patients in the placebo group (p = 0.39). The absolute 6MWD decreased from baseline to week 12 in both groups. However, some additional endpoints such as oxygenation, diffusion capacity for carbon monoxide, Borg dyspnoea index and quality of life scores were slightly but significantly improved. The primary endpoint of STEP-IPF would have been met by 31% of the PH-IIP patients in our series, raising the possibility that patients with IIP and PH may be more likely to respond to pulmonary vasodilator therapy than unselected patients with interstitial lung disease.

During the last World Symposium on Pulmonary Hypertension, held 2013 in Nice, France, a working group addressed the use of pulmonary vasodilator therapy in patients with lung disease and pulmonary hypertension according to the severity of both components [3]. Severe PH was defined by a PAPm ≥35 mmHg or a PAPm ≥25 mmHg with a cardiac index below 2.0 L/min/m^2^ at rest. This definition was not evidence-based and our data do not support the notion that patients with severe PH have a better clinical response to pulmonary vasodilator therapy than other patients with PH-IIP.

Our study has several strengths and limitations. Strengths include the relatively large cohort of prospectively enrolled and consecutive patients newly diagnosed with pulmonary hypertension by right heart catheterization who were followed for a period of up to 5 years. Our cohort of patients with PH-IIP receiving pulmonary vasodilator therapy is one of the largest of its kind to date. The number of patients lost to follow-up was less than 3% in both groups. In addition, the study included a large control group of patients with IPAH treated at the same centres during the same time period. The initial responses to treatment as well as the survival rates observed in the IPAH patients match the effects seen in clinical trials and large registries, perhaps lending some credibility to the treatment effects observed in the PH-IIP cohort. Limitations include the paucity of data on the underlying parenchymal lung disease. Data on lung function and blood gases were available and all centres used a multidisciplinary approach to determine the underlying lung disease, but detailed data on CT findings and histologies were not captured in the database. Additional limitations include missing values for some of the variables, the lack of systematic follow-up assessments on haemodynamics, lung function and blood gases, the fact that the majority of the patients came from Germany limiting the generalizability of the findings, and–most importantly–the lack of a control group of patients with PH-IIP who did not receive targeted medical therapy.

In conclusion, we describe a population of patients with chronic fibrotic IIP characterized by mostly severe PH. These patients were treated with pulmonary vasodilators; predominantly PDE5i, and our data indicate some early improvements in 6MWD and functional class that were comparable to those seen in patients with IPAH. The overall survival of patients with PH-IIP was significantly worse than the survival of patients with IPAH. These findings underscore the need to conduct randomized long-term trials addressing safety and tolerability of pulmonary vasodilator therapies as well as their effects on exercise-capacity, quality of life and long-term outcomes in patients with lung disease and PH.
